# Supplementary material for: Targeted Screening of Lactiplantibacillus plantarum Strains Isolated from Tomatoes and Its Application in Tomato Fermented Juice
Source: Foods. 2024 Nov 8;13(22):3569. doi: 10.3390/foods13223569 (PMC11593249; doi:10.3390/foods13223569)
Supplement: Supplementary file 1 [file foods-13-03569-s001.zip › foods-3263882-supplementary.pdf]

Supplementary Materials

Targeted Screening of *Lactiplantibacillus plantarum* Strains Isolated from Tomatoes and Its Application in Tomato Fermented Juice

Nuersiman Tuerhong [1,†], Liang Wang [1,†], Jie Cui [2], Dilireba Shataer [1], Huizhen Yan [1], Xiaoxiao Dong [1], Ziqi Gao [1], Minwei Zhang [1], Yanan Qin [1] and Jing Lu [1,*]

[1] Xinjiang Key Laboratory of Biological Resources and Genetic Engineering, College of Life Science and Technology, Xinjiang University, Urumqi 830046, China; 15099330343@163.com (N.T.); wl1390593786@163.com (L.W.); dlrb_shataer2021@xju.edu.cn (D.S.); 18536983054@163.com (H.Y.); 15599675517@163.com (X.D.); gaoziqi666@sina.com (Z.G.); zhangmw@xju.edu.cn (M.Z.); qingyalan12345@sina.com (Y.Q.)

[2] College of Food Science and Light Industry, Nanjing Tech University, Nanjing 211816, China; jiecui_njtech@163.com

* Correspondence: jinglu@xju.edu.cn

† These authors contributed equally to this work.

Table S1. Analysis of Basic Physiological Characteristics of Lactic Acid Bacteria From Tomato Enzyme Source

Strain Number	Gram Strining	Catalase	Indole	Gelatin Liquefaction	Carbon Dioxide Gas production
A1	+	-	+	+	-
A2	+	-	+	+	-
A3	+	-	+	+	-
A4	+	-	+	+	-
A5	+	-	+	+	-
A6	+	-	+	+	-
A7	+	-	+	+	-
A8	+	-	+	+	-
A9	+	-	+	+	-
A10	+	-	+	+	-
A11	+	-	+	+	-
A12	+	-	+	+	-
A13	+	-	+	+	-
A14	+	-	+	+	-
A15	+	-	+	+	-
A16	+	-	+	+	-
A17	+	-	+	+	-
A18	+	-	+	+	-
A19	+	-	+	+	-
A20	+	-	+	+	-
A21	+	-	+	+	-

A22	+	-	+	+	-
A23	+	-	+	+	-
A24	+	-	+	+	-
A25	+	-	+	+	-
A27	+	-	+	+	-
A28	+	-	+	+	-
A29	+	-	+	+	-
A30	+	-	+	+	-
A31	+	-	+	+	-
A32	+	-	+	+	-
A33	+	-	+	+	-
A34	+	-	+	+	-
A35	+	-	+	+	-
A36	+	-	+	+	-
A37	+	-	+	+	-
